# Heterogeneity of Age and Its Associated Features in Patients with Critical Limb Ischemia

**DOI:** 10.3400/avd.oa.20-00067

**Published:** 2020-09-25

**Authors:** Mitsuyoshi Takahara, Osamu Iida, Yoshimitsu Soga, Akio Kodama, Hiroto Terashi, Kenji Suzuki, Ikuo Sugimoto, Nobuyoshi Azuma

**Affiliations:** 1 Department of Metabolic Medicine, Osaka University Graduate School of Medicine; 2 Department of Diabetes Care Medicine, Osaka University Graduate School of Medicine; 3 Cardiovascular Center, Kansai Rosai Hospital; 4 Department of Cardiology, Kokura Memorial Hospital; 5 Division of Vascular Surgery, Department of Surgery, Nagoya University Graduate School of Medicine; 6 Department of Plastic Surgery, Kobe University Graduate School of Medicine; 7 Department of Cardiology, Tokyo Saiseikai Central Hospital; 8 Department of Vascular Surgery, Aichi Medical University; 9 Department of Vascular Surgery, Asahikawa Medical University

**Keywords:** critical limb ischemia, age distribution, cardiovascular risk factor, socioeconomic status

## Abstract

**Aim**: Critical limb ischemia (CLI) has a wide age distribution. We aimed here to reveal age-associated clinical features in CLI patients.

**Materials and Methods**: We analyzed 531 Japanese CLI patients referred to vascular centers. The three-year mortality risk by age was compared to that for the Japanese nationals, derived from Japan’s national life table data. Clinical characteristics associated with age in CLI patients were also explored.

**Results**: Mean age was 73±10 years. Whereas 27.9% were aged ≥80 years, 19.2% were aged <65 years. Mortality risk was increased with age, but its risk ratio relative to the same-aged nationals was higher in younger patients. Incidence of major amputation was higher in a younger population. Receiving welfare, smoking, increased body mass index, diabetes with hemoglobin A1c ≥7.0%, non-high density lipoprotein cholesterol ≥190 mg/dL, renal failure, and the Wound, Ischemia, and foot Infection classification stage 4 were associated with younger age, whereas non-ambulation and institutionalization were associated with older age.

**Conclusion**: Patients aged <65 years, belonging to the working-age population, reached almost one fifth of the CLI population. Younger patients had a lower mortality risk in the population, but had a higher risk ratio relative to the same-aged nationals. Socioeconomic disadvantage, poor cardiovascular risk control, and wound severity were associated with younger age.

## Introduction

Critical limb ischemia (CLI) is the most advanced form of peripheral arterial disease, which is characterized as rest pain or skin lesions (either ulcers or gangrenes), due to chronic severe ischemia.^1)^ It is well recognized that the mean age of a CLI population is around 70 to 75 years,^2–4)^ and is evidently higher than that of a non-CLI population.^5)^ On the other hand, the distribution range is considerably wide (about 35 to 100 years),^5)^ meaning that the population is heterogeneous in age, and that young CLI patients are not rare in clinical practice. However, clinical features associated with the age heterogeneity remained unrevealed. Clinical problems, e.g., metabolic and nutritional control, as well as wound severity, would sometimes need to be addressed differently by age group, adopting different strategies and utilizing different resources. Needs for rehabilitation back into society would also vary with age. For cardiovascular centers to take proper measures for CLI patients, a clear understanding of the link between their clinical features and age would be important. Furthermore, the elderly and the nonelderly are generally supported by different health care and social security systems. For the insurers and the authorities, the information on age-specific features in the population would be also of use. The aim of the current study was therefore to reveal clinical features that were associated with age in CLI patients.

## Materials and Methods

We used a clinical database obtained from the Surgical reconstruction versus Peripheral INtervention in pAtients with critical limb isCHemia (SPINACH) study, a prospective, multicenter, observational study that registered patients who had CLI due to atherosclerotic arterial disease in 23 centers (12 vascular surgery departments and 11 interventional cardiology departments) in Japan.^6,7)^ CLI patients were registered at the referral to the participating centers, between January 2012 and March 2013. The details of the SPINACH study are described elsewhere.^6,7)^ The study was performed in accordance with the Declaration of Helsinki and was approved by the ethics committee at the principal research institution, the Asahikawa University Hospital (no. 1023), and all the other centers registering patients. Written informed consent was obtained. The current analysis included a total of 531 patients presenting either ischemic wound with the Wound, Ischemia, and foot Infection (WIfI) classification system^8)^ I-2/3 or ischemic rest pain with the WIfI I-3. Skin perfusion pressures of 31–40 mmHg and ≤30 mmHg were treated as WIfI I-2 and 3, respectively.^7)^ Non-ambulatory status was determined when patients were on a wheelchair or bed-ridden. Body mass index (BMI) was classified into <18.5 (lean), 18.5 to 25, and ≥25 kg/m^2^ (obese). Hemoglobin A1c (HbA1c) levels in diabetic patients were categorized into <7%, 7% to 8%, and ≥8%.^9)^ Blood pressure was classified into <130/80, 130/80 to 140/90, 140/90 to 160/100, and ≥160/100 mmHg.^10)^ Reduced high-density lipoprotein cholesterol (HDLC) levels were determined as <40 mg/dL, whereas non-HDLC levels, calculated as total cholesterol levels minus HDLC levels, were categorized into <100, 100 to 130, 130 to 170, 170 to 190, and ≥190 mg/dL.^11)^ Renal failure was defined as estimated glomerular filtration rate <30 mL/min/1.73 m^2^ or requirement of dialysis.

### Statistical analysis

Baseline data are given as means and standard deviations for continuous variables or as frequencies and percentages for discrete variables. A P-value of <0.05 was considered statistically significant, and 95% confidence intervals are reported when appropriate. Descriptive statistics are demonstrated in the overall population, and in subgroups aged <65 years, 65 to 79 years, and ≥80 years. Difference in age among treatment strategies was tested by the Welch’s one-way analysis of variance. The three-year cumulative incidence rate of mortality was estimated using the Kaplan–Meier method, whereas that of major amputation was estimated using the cumulative incidence function while treating mortality as competing risk. The association of age with the three-year risk of mortality and major amputation in the study population was evaluated using the Cox proportional hazards regression model. For the analysis of major amputation, the Fine-Gray competing risk model adjusted for mortality risk was employed.The impact of treatment strategies on the risk of mortality and major amputation was evaluated by the analysis of variance for a Cox model. Time-to-event regression models were developed only when ten or more events were observed. The three-year cumulative incidence rate by age was estimated by the Cox model using the smoothing spline. All of these time-to-event analyses were performed using the R package survival. The three-year cumulative incidence rate of mortality was further compared to that of the sex-adjusted Japanese nationals in 2012, which was derived from the Japan’s national life table data published by the Ministry of Health, Labor, and Welfare. ^12)^ For the adjustment for sex, the smoothing spline of the sex distribution by age in the CLI population was estimated by the generalized additive model, using the R package mgcv. The 95% confidence intervals of the three-year cumulative incidence rates of mortality and their risk ratios relative to Japanese nationals were obtained from the 2,000-time bootstrapping method. We subsequently explored the association of clinical features with age, using the linear regression model. Missing data were addressed using the multiple imputation (10 times) by chained equations method, using the R package mice. All statistical analyses were performed using R version 3.6.0 (R Development Core Team, Vienna, Austria).

## Results

**Figure 1:**
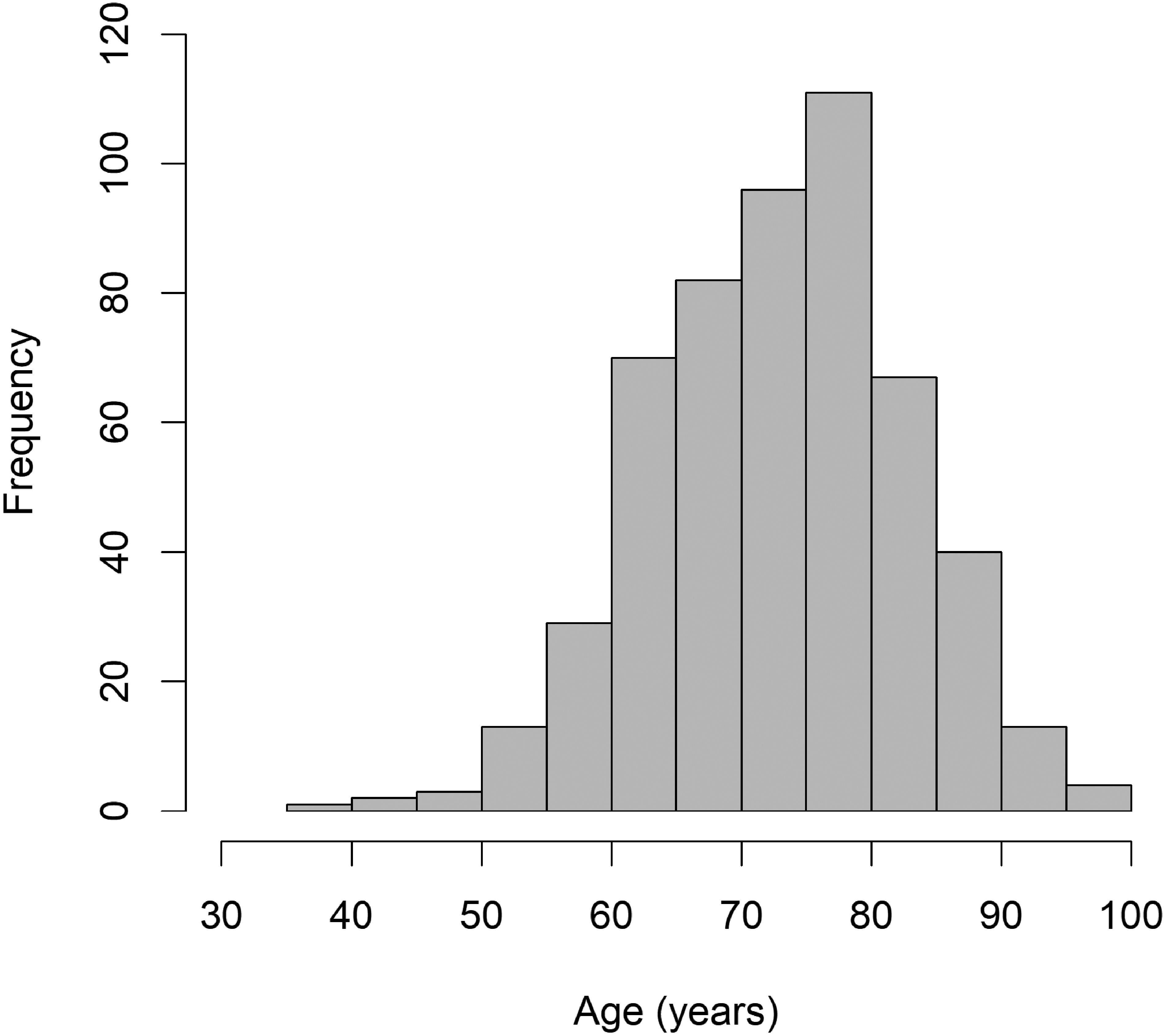
Fig. 1 Histogram of age in the study population.

Background characteristics of the study population with CLI are shown in **Table 1**. The mean age was 73±10 years old. Age was ranged from 39 to 100 years (**Fig. 1**). A total of 102 patients (19.2%) were younger than 65 years, whereas 148 (27.9%) were 80 years or older. The majority of the study population were patients undergoing endovascular therapy without prior history of revascularization for index CLI (i.e., primary endovascular therapy) (n=313) and those undergoing surgical reconstruction without prior history of revascularization for index CLI (i.e., primary surgical reconstruction) (n=129) (**Table 2**).

**Table 1:** Table 1 Characteristics of the CLI study population

	Overall population (n=531)	Patients aged <65 years (n=102)	Patients aged 65 to 79 years (n=281)	Patients aged ≥80 years (n=148)
Age (years)	73±10	59±5	73±4	85±4
Sex				
Male sex	355 (66.9%)	73 (71.6%)	203 (72.2%)	79 (53.4%)
Female sex	176 (33.1%)	29 (28.4%)	78 (27.8%)	69 (46.6%)
Ambulatory status				
Ambulatory	279 (52.5%)	64 (62.7%)	151 (53.7%)	64 (43.2%)
Non-ambulatory	252 (47.5%)	38 (37.3%)	130 (46.3%)	84 (56.8%)
Body mass index				
<18.5 kg/m^2^ (lean)	85 (16.0%)	9 (8.8%)	41 (14.6%)	35 (23.6%)
18.5 to 25 kg/m^2^	351 (66.1%)	62 (60.8%)	197 (70.1%)	92 (62.2%)
≥25 kg/m^2^ (obase)	95 (17.9%)	31 (30.4%)	43 (15.3%)	21 (14.2%)
Living place				
Living at home	493 (92.8%)	101 (99.0%)	269 (95.7%)	123 (83.1%)
Staying in nursing home	38 (7.2%)	1 (1.0%)	12 (4.3%)	25 (16.9%)
Receiving welfare				
No	479 (90.2%)	84 (82.4%)	257 (91.5%)	138 (93.2%)
Yes	52 (9.8%)	18 (17.6%)	24 (8.5%)	10 (6.8%)
Smoking				
Never	214 (40.3%)	29 (28.4%)	95 (33.8%)	90 (60.8%)
Past	233 (43.9%)	46 (45.1%)	143 (50.9%)	44 (29.7%)
Current	84 (15.8%)	27 (26.5%)	43 (15.3%)	14 (9.5%)
Diabetes mellitus				
Non-diabetes	142 (26.9%)	23 (22.5%)	64 (22.9%)	55 (37.7%)
Diabetes with HbA1c <7%	254 (48.2%)	45 (44.1%)	139 (49.8%)	70 (47.9%)
Diabetes with HbA1c 7% to 8%	75 (14.2%)	17 (16.7%)	47 (16.8%)	11 (7.5%)
Diabetes with HbA1c ≥8%	56 (10.6%)	17 (16.7%)	29 (10.4%)	10 (6.8%)
Blood pressure				
<130/80 mmHg	183 (34.7%)	25 (25.0%)	102 (36.4%)	56 (38.1%)
130/80 to 140/90 mmHg	109 (20.7%)	20 (20.0%)	62 (22.1%)	27 (18.4%)
140/90 to 160/100 mmHg	133 (25.2%)	34 (34.0%)	61 (21.8%)	38 (25.9%)
≥160/100 mmHg	102 (19.4%)	21 (21.0%)	55 (19.6%)	26 (17.7%)
Non-HDLC				
<100 mg/dL	205 (41.2%)	30 (31.9%)	125 (47.5%)	50 (35.7%)
100–130 mg/dL	144 (29.0%)	29 (30.9%)	69 (26.2%)	46 (32.9%)
130–170 mg/dL	111 (22.3%)	28 (29.8%)	51 (19.4%)	32 (22.9%)
170–190 mg/dL	20 (4.0%)	3 (3.2%)	10 (3.8%)	7 (5.0%)
≥190 mg/dL	17 (3.4%)	4 (4.3%)	8 (3.0%)	5 (3.6%)
HDLC <40 mg/dL	208 (40.7%)	46 (47.4%)	112 (41.0%)	50 (35.5%)
Renal failure				
No	218 (41.1%)	31 (30.4%)	108 (38.4%)	79 (53.4%)
Yes	313 (58.9%)	71 (69.6%)	173 (61.6%)	69 (46.6%)
Heart failure				
No	431 (81.2%)	81 (79.4%)	230 (81.9%)	120 (81.1%)
Yes	100 (18.8%)	21 (20.6%)	51 (18.1%)	28 (18.9%)
Coronary artery disease				
No	311 (58.6%)	58 (56.9%)	155 (55.2%)	98 (66.2%)
Yes	220 (41.4%)	44 (43.1%)	126 (44.8%)	50 (33.8%)
Ischemic stroke				
No	423 (79.7%)	83 (81.4%)	219 (77.9%)	121 (81.8%)
Yes	108 (20.3%)	19 (18.6%)	62 (22.1%)	27 (18.2%)
WIfI classification				
Clinical stage 2	91 (17.1%)	8 (7.8%)	60 (21.4%)	23 (15.5%)
Clinical stage 3	154 (29.0%)	28 (27.5%)	76 (27.0%)	50 (33.8%)
Clinical stage 4	286 (53.9%)	66 (64.7%)	145 (51.6%)	75 (50.7%)

Data are mean±standard deviation (SD), or frequency (percentage). Data on HbA1c, blood pressure, non-HDLC, and HDLC were missing in 4 (0.8%), 4 (0.8%), 34 (6.4%), and 20 (3.8%), respectively. The other variables than HbA1c, blood pressure, non-HDLC, and HDLC had no missing data.CLI: critical limb ischemia; HDL: high-density lipoprotein; HbA1c: hemoglobin A1c; WIfI: Wound, Ischemia, and foot Infection classification

**Table 2:** Table 2 Age and risk of mortality and major amputation by revascularization strategy

	n	Age (yrs)	3-year mortality risk				3-year major amputation risk			
			No. of observed events	Cumulative incidence rate	Hazard ratio of age (per 10 yrs)	P*^1)^	No. of observed events	Cumulative incidence rate	Hazard ratio of age (per 10 yrs)	P*^1)^
Overall	531	73±10	232	48% [43%–53%]	1.42 [1.24–1.63]	—	46	9% [7%–12%]	0.73 [0.55–0.97]	—
EVT, primary	313	74±10	137	49% [43%–55%]	1.42 [1.19–1.70]	(Ref)	24	8% [6%–12%]	0.81 [0.54–1.21]	(Ref)
EVT, redo	16	74±8	8	63% [21%–82%]	—	—	2	13% [3%–46%]	—	—
Surgical, primary	129	73±9	46	37% [28%–45%]	1.52 [1.09–2.11]	0.73	13	11% [6%–18%]	0.49 [0.28–0.86]	0.16
Surgical, redo	22	70±7	8	41% [14%–60%]	—	—	3	14% [5%–39%]	—	—
Hybrid, primary	34	71±11	21	66% [44%–79%]	1.29 [0.87–1.92]	0.65	2	6% [2%–23%]	—	—
Hybrid, redo	3	69±7	2	33% [0%–70%]	—	—	0	0% [N/A]	—	—
Conservative	14	82±12	10	77% [32%–92%]	1.23 [0.73–2.08]	0.68	2	14% [4%–52%]	—	—
P*^2)^ (crude)	—	0.083	—	0.032	—	—	—	0.81	—	—
P*^2)^ (age-adjusted)	—	—	—	0.29	—	—	—	0.88	—	—

Data are means±standard deviations for age, and estimates [95% confidence intervals] for cumulative incidence rates and hazard ratios. P*^1)^: P-values for the difference in the hazard ratio of age vs. primary EVT; P*^2)^: P-values for the difference among treatment strategies. Hazard ratios were calculated only when 10 or more events were observed. “Primary” revascularization indicates revascularization without prior history of revascularization for index CLI, whereas “redo” revascularization indicates revascularization after prior revascularization for index CLI.Conservative: conservative therapy without any revascularization; EVT: endovascular therapy; Hybrid: hybrid therapy of endovascular therapy and surgical reconstruction; Surgical: surgical reconstruction; N/A: not applicable (unable to be estimated) due to no observed events; CLI: critical limb ischemia; HDL: high-density lipoprotein; HbA1c: hemoglobin A1c; WIfI: Wound, Ischemia, and foot Infection classification

**Figure 2:**
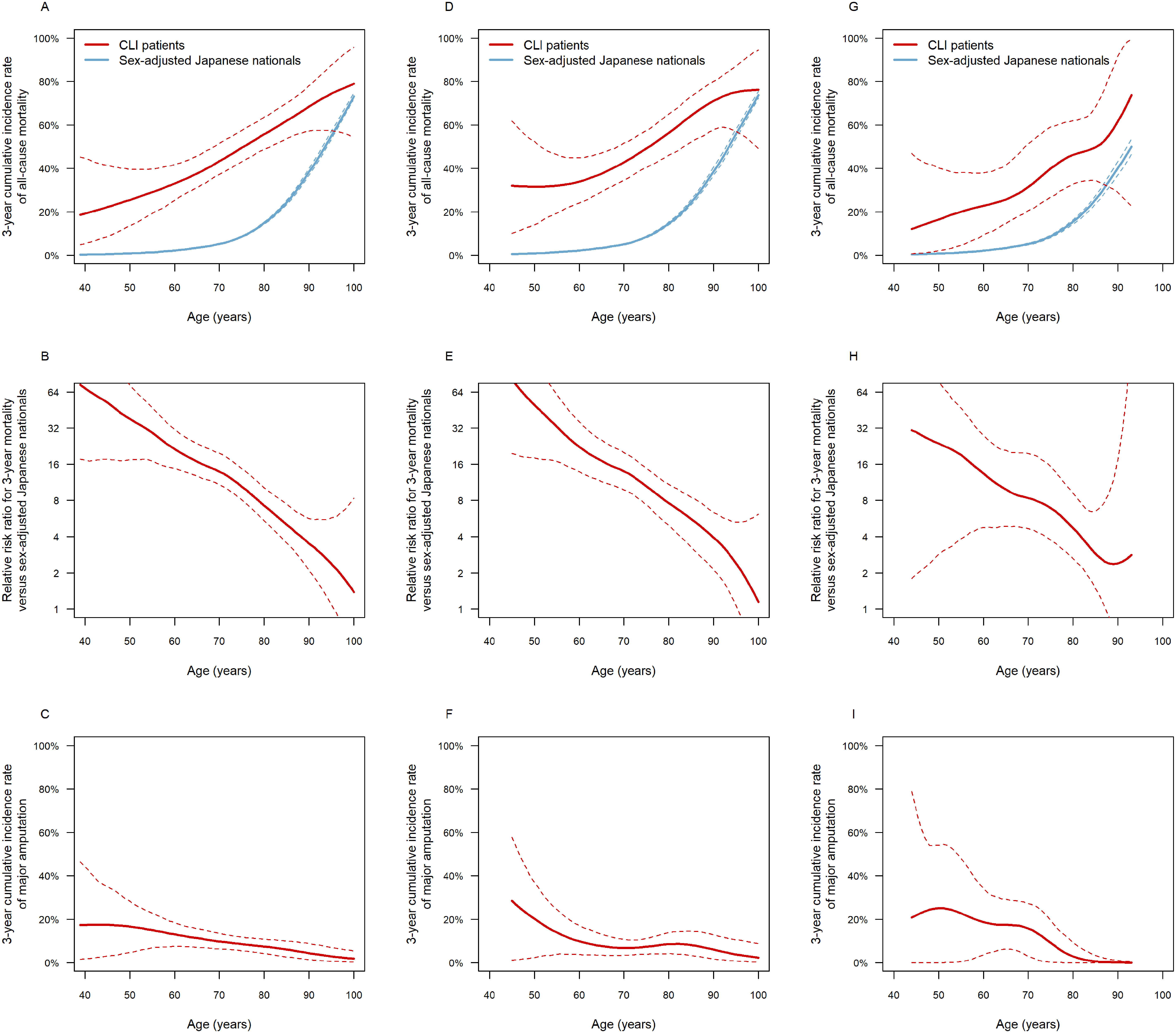
Fig. 2 Mortality risk by age in the CLI study population and comparison with sex-adjusted nationals.

As illustrated in **Table 2** and **Fig. 2A**, the three-year mortality risk was increased with age in the CLI population. On the contrary, when compared to the sex-adjusted nationals of the same age, its relative risk ratio was higher in a younger age group (**Fig. 2B**). Age was inversely associated with the risk of major amputation (**Table 2** and **Fig. 2C**). Similar trends were observed in patients undergoing primary endovascular therapy and those undergoing primary surgical reconstruction (**Table 2** and **Figs. 2D**–**2I**).

Data are estimates (bold lines) and 95% confidence intervals (dashed lines) in the overall population (n=531) (**Panel A** to **C**), patients undergoing primary endovascular therapy (n=313) (**Panel D** to **F**), and those undergoing primary surgical reconstruction (n=129) (**Panel G** to **I**). **Panel A**, **D**, and **G** show the 3-year cumulative incidence rate of all-cause mortality corresponding to ages in the CLI study population (red lines) and the sex-adjusted nationals derived from the life table data of the Japanese nationals (blue lines). **Panel B**, **E**, and **H** show the risk ratio for the 3-year mortality relative to the sex-adjusted nationals of the same age in the CLI study population. **Panel C**, **F**, and **I** show the 3-year cumulative incidence rate of major amputation corresponding to ages in the CLI study population.

**Table 3** demonstrates the association of clinical features with age. Increased BMI, receiving welfare, smoking, diabetes mellitus with HbA1c ≥7.0%, non-HDLC levels ≥190 mg/dL, renal failure, and WIfI clinical stage 4 were inversely associated with age. The corresponding age difference was −2.2 [−4.3 to 0.0] years (P=0.048) for BMI of 18.5 to 25 kg/m^2^, −5.5 [−8.2 to −2.9] years (P<0.001) for BMI of ≥25 kg/m^2^, −3.6 [−6.1 to −1.0] years (P=0.006) for receiving welfare, −3.2 [−5.1 to −1.2] years (P=0.001) for past smoking, −5.8 [−8.3 to −3.3] years (P<0.001) for current smoking, −3.3 [−5.7 to −0.8] years (P=0.010) for diabetes mellitus with HbA1c 7% to 8%, −5.1 [−7.9 to −2.4] years (P<0.001) for diabetes mellitus with HbA1c ≥8%, −5.4 [−10.1 to −0.8] years (P=0.022) for non-HDLC levels ≥190 mg/dL, −3.5 [−5.1 to −1.8] years (P<0.001) for renal failure, and −2.8 [−4.9 to −0.7] years (P=0.008) for WIfI clinical stage 4, respectively. On the other hand, non-ambulatory status and staying in a nursing home were positively associated with age; the corresponding age difference was 2.0 [0.4 to 3.6] years (P=0.015) and 5.5 [2.4 to 8.6] years (P<0.001), respectively. In other words, self-ambulatory status and not staying in a nursing home were associated with younger age.

**Table 3:** Table 3 Clinical features associated with age in the CLI study population

	Crude regression coefficient	Adjusted regression coefficient
Male sex	−3.9 [−5.7 to −2.2] (P<0.001)	−1.6 [−3.4 to 0.3] (P=0.10)
Non-ambulatory status	3.2 [1.5 to 4.8] (P<0.001)	2.0 [0.4 to 3.6] (P=0.015)
Body mass index (versus <18.5 kg/m^2^)		
18.5 to 25 kg/m^2^	−3.7 [−6.0 to −1.5] (P=0.001)	−2.2 [−4.3 to 0.0] (P=0.048)
≥25 kg/m^2^	−7.3 [−10.1 to −4.5] (P<0.001)	−5.5 [−8.2 to −2.9] (P<0.001)
Staying in nursing home	8.9 [5.8 to 12.0] (P<0.001)	5.5 [2.4 to 8.6] (P<0.001)
Receiving welfare	−3.8 [−6.6 to −1.0] (P=0.007)	−3.6 [−6.1 to −1.0] (P=0.006)
Smoking (versus never)		
Past	−4.6 [−6.3 to −2.9] (P<0.001)	−3.2 [−5.1 to −1.2] (P=0.001)
Current	−7.7 [−10.0 to −5.3] (P<0.001)	−5.8 [−8.3 to −3.3] (P<0.001)
Diabetes mellitus (versus nondiabetes)		
Diabetes with HbA1c <7%	−2.6 [−4.5 to −0.6] (P=0.009)	−0.7 [−2.6 to 1.2] (P=0.47)
Diabetes with HbA1c 7% to 8%	−4.9 [−7.6 to −2.2] (P<0.001)	−3.3 [−5.7 to −0.8] (P=0.010)
Diabetes with HbA1c ≥8%	−7.0 [−9.9 to −4.0] (P<0.001)	−5.1 [−7.9 to −2.4] (P<0.001)
Blood pressure (versus <130/80 mmHg)		
130/80 to 140/90 mmHg	−1.5 [−3.9 to 0.8] (P=0.20)	−1.3 [−3.4 to 0.7] (P=0.20)
140/90 to 160/100 mmHg	−1.9 [−4.1 to 0.2] (P=0.082)	−1.3 [−3.3 to 0.7] (P=0.20)
≥160/100 mmHg	−2.0 [−4.3 to 0.4] (P=0.10)	−0.7 [−2.9 to 1.5] (P=0.52)
Non-HDLC (versus <100 mg/dL)		
100−130 mg/dL	0.0 [−2.0 to 2.1] (P=0.96)	−0.6 [−2.4 to 1.3] (P=0.54)
130−170 mg/dL	−1.0 [−3.2 to 1.2] (P=0.36)	−2.0 [−4.1 to 0.0] (P=0.055)
170−190 mg/dL	0.5 [−4.0 to 5.0] (P=0.83)	−1.5 [−5.5 to 2.6] (P=0.49)
≥190 mg/dL	−2.1 [−7.5 to 3.3] (P=0.43)	−5.4 [−10.1 to −0.8] (P=0.022)
HDLC <40 mg/dL	−1.7 [−3.5 to 0.0] (P=0.055)	0.0 [−1.7 to 1.6] (P=0.97)
Renal failure	−3.4 [−5.1 to −1.7] (P<0.001)	−3.5 [−5.1 to −1.8] (P<0.001)
Heart failure	0.3 [−1.9 to 2.4] (P=0.81)	−0.2 [−2.1 to 1.8] (P=0.87)
Coronary artery disease	−1.8 [−3.5 to −0.1] (P=0.033)	−0.7 [−2.3 to 0.9] (P=0.39)
Ischemic stroke	−0.2 [−2.2 to 1.9] (P=0.88)	−0.1 [−2.0 to 1.8] (P=0.92)
WIfI classification (versus stage 2)		
Clinical stage 3	−0.9 [−3.4 to 1.6] (P=0.48)	−0.5 [−2.8 to 1.8] (P=0.67)
Clinical stage 4	−2.9 [−5.2 to −0.6] (P=0.014)	−2.8 [−4.9 to −0.7] (P=0.008)

Data are regression coefficients and 95% confidence intervals (P-values). Crude regression coefficients were derived from respective univariate linear regression models, whereas adjusted regression coefficients were from the multivariate linear regression model in which all the explanatory variables listed in the table were entered.CLI: critical limb ischemia; HDL: high-density lipoprotein; HbA1c: hemoglobin A1c; WIfI: Wound, Ischemia, and foot Infection classification

**Figure 3:**
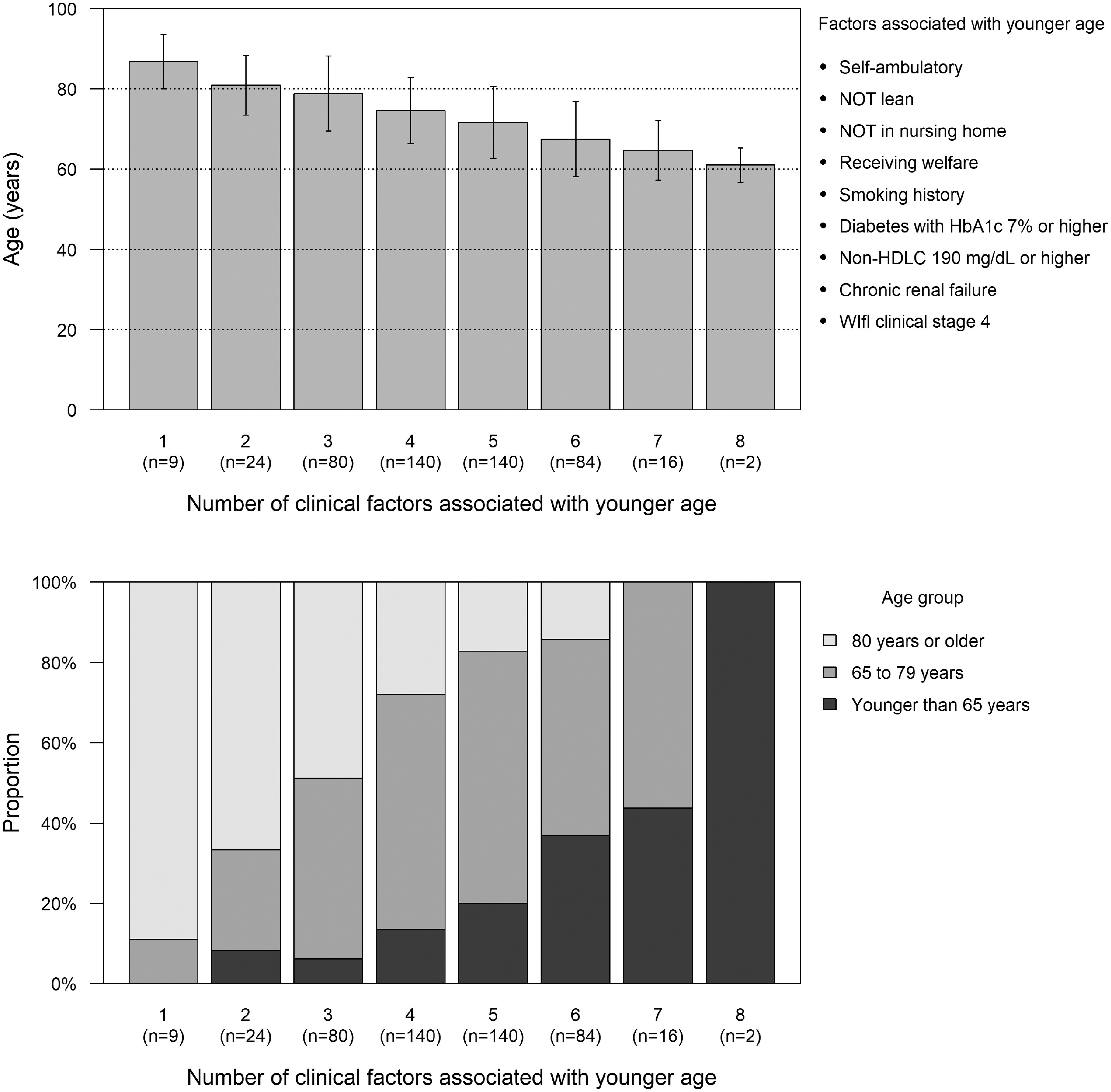
Fig. 3 Age by accumulation of clinical factors associated with younger age.

These clinical factors were associated with age independently of one another, suggesting that their impact on age was additive. Indeed, as shown in **Fig. 3**, age distribution was younger when more of the following clinical features were accumulated: (1) self-ambulatory status; (2) not being lean; (3) not staying in a nursing home; (4) receiving welfare; (5) having smoking history; (6) diabetes mellitus with HbA1c ≥7%; (7) non-HDLC ≥190 mg/dL; (8) renal failure; and (9) WIfI clinical stage 4. Mean age was almost 90 years in patients with only one of these factors, whereas it was about 60 years in patients with eight of these accumulated factors.

Data are means and standard deviations of age (upper panel) and the proportion of patients aged ≥80 years, 65 to 79 years, and <65 years (lower panel) in the subgroups of the CLI study population classified according to the number of clinical factors associated with younger age.

## Discussion

The current study demonstrated clinical features associated with the heterogeneity of age in CLI patients. Patients aged <65 years reached almost one fifth of the CLI population. Younger patients developing CLI had a lower mortality risk than older patients, but the risk ratio relative to the sex-adjusted nationals of the same age was higher in younger patients. Furthermore, the incidence of major amputation was higher in a younger population. Socioeconomic disadvantage, poor cardiovascular risk control, and wound severity were associated with younger age, whereas frail aspects were likely associated with older age.

CLI patients are often described as an older population on average than non-CLI patients.^5)^ Indeed, more than a quarter was aged 80 years or older in this current study population. On the other hand, almost one fifth were younger than 65 years, belonging administratively to the working-age population. Young CLI patients were not rare in clinical practice. In the CLI population, the mortality risk was linearly increased with age. This positive relationship in CLI patients was consistent with a number of previous reports,^13)^ and would be no surprise. However, the subsequent comparison with the national standard value clarified the fact that younger patients had an extremely higher risk ratio of mortality relative to the nationals of the same age. Patients developing CLI younger might be a subgroup with a lower mortality risk within the CLI population, but suffered more greatly from the survival disparity relative to the same generation in the nationals.

The multivariate linear regression analysis revealed that non-ambulatory status, leanness, and stay in a nursing home were positively related to increased age. Elderly people often have geriatric health problems, and one of the major problems is frailty.^14)^ It would be no surprise that old CLI patients were likely to have these clinical features.

By contrast, smoking history, diabetes mellitus with elevated HbA1c levels, high non-HDLC levels, and renal failure, as well as obesity, were inversely associated with age. All these factors are well known as major accelerators of atherosclerosis, or vascular aging. The current inverse correlations would indicate that patients with accumulated cardiovascular risk factors will develop CLI earlier (i.e., at a younger age), whereas those with fewer will develop the disease later (i.e., at an older age).^15)^

Another factor inversely associated with age was receiving welfare. The literature suggest that low socioeconomic status would be linked to the development of cardiovascular diseases and may confer a cardiovascular risk that is equivalent to traditional cardiovascular risk factors.^16)^ Its link would be partially explained by the correlation with diet and lifestyles.^17)^ Patients with low socioeconomic status, more likely spending unhealthy lives, might accelerate atherosclerosis, and develop CLI at younger age.

WIfI clinical stage 4 was also associated with younger age. Compared to older patients, younger patients might be less sedentary, and more likely keep their dynamic activities in daily lives, including walking, after foot ulceration. Such weight-bearing activities would expose the index foot to heavy mechanical loads, which might deteriorate ulcers.^18)^ Furthermore, mobilization might deteriorate infection.^19)^ A higher incidence of major amputation in a younger population might reflect the fact that they presented severer CLI, together with poorer cardiovascular risk control. The developmental course of CLI might be different between younger and older patients.

The current study had some limitations. Firstly, the number of patients undergoing treatment strategies other than primary endovascular therapy and primary surgical reconstruction were so small that the prognosis of these populations and their association with age remained unclear. The difference among treatment strategies, yielding no statistical significance, would be also inconclusive. Furthermore, the number of observed events was limited, and multivariate risk analyses with adjustment for multiple covariates were not performed. Secondly, the current analysis only demonstrated a cross-sectional relationship between clinical features and age. Although the association of clinical profiles with age is suggestive of mechanisms of CLI onset, the causal relationships between these clinical profiles and CLI onset remained unrevealed. Longitudinal studies will be needed to reveal the involvement of these clinical features in CLI development. Thirdly, the current study analyzed Japanese CLI patients. It remained unknown whether similar findings would be observed in other ethnic populations.

## Conclusion

Patients aged <65 years, belonging to the working-age population, reached almost one fifth of the CLI population. Patients developing CLI younger had a lower mortality risk than older patients, but the risk ratio relative to the sex-adjusted nationals of the same age was higher in younger patients. Incidence of major amputation was higher in a younger population. Socioeconomic disadvantage, poor cardiovascular risk control, and wound severity were associated with younger age, whereas frail aspects were likely associated with older age.
